# Hospital Meetings

**Published:** 1901-03-09

**Authors:** 


### The Institutional Workshop.

### HOSPITAL MEETINGS.

### THE MIDDLESEX HOSPITAL.

#### Cancer Investigation and Treatment.

Lord Sandhurst presided at the quarterly Court of Governors of the Middlesex Hospital on February 28th, when the annual report and accounts were presented. In the report, which was read by the secretary-superintendent, Mr. Clare Melhado, the board first expressed their sense of the loss sustained by the nation by the death of her late Majesty, and recorded the fact that a special Court of Governors held for the purpose, with the president of the hospital, the Duke of Northumberland, in the chair, had presented an address to the King expressing sympathy in his bereavement and congratulation on his accession.

Turning to the work of the hospital, the board directed attention to the removal of the female cancer patients to the new wing erected for their accommodation, and to the fact that they had in consequence decided upon separating the statistics and accounts relative to all cancer patients and the Cancer Investigation Department from those of the general side of the hospital. Owing to this no comparison was possible in the figures with those of former years, and the same applied to the index number, found by multiplying the number of medical and surgical in-patients by their respective average stay and adding the results. For 1890 the index number thus obtained was 93,723. In arriving at the ratio of expenses of management to total ordinary expenditure, the generally-recognised test of efficiency, it was also necessary to take into consideration the accounts of the Cancer Fund, which included a merely nominal charge for management. On this basis the figures showed the ratio to be less than 5 per cent., although a greater number of patients had been treated and the ordinary expenditure increased, and although no account was taken of the convalescent patients, receiving benefits from the Samaritan Fund and convalescent home.

The number of in-patients treated during the year was 3,758. Of these 180 were cancer patients. The out-patients numbered 48,911, and their attendances 113,373. The average weekly cost of each in-patient was £1 18s. 0⅜d., and for each out-patient 1s. 4⅝d. The cost of each occupied bed for the year was £82 2s. 8½d. The average stay in hospital was, for medical patients 28·69 days, surgical patients 24·3 days, cancer patients 89 days. The average daily number of beds occupied was 293. Of the convalescent patients 715 received additional relief at the Home at Clacton-on-Sea.

Taking the general account separately from those of the Cancer and Cancer Investigation Funds, the ordinary income was £15,772, and the extraordinary income £15,507, made up of legacies £13,207, endowments £2,050, and a special grant of £250 from the Prince's Fund, made upon conditions now receiving the consideration of the Board and Medical Committee. The ordinary expenditure was £28,925, £1,036 having been deducted as the cost of the nine male cancer patients still remaining in the main building and charged to the Cancer Fund; the extraordinary expenditure was £5,396 for building improvements, plus £3,000 contributed towards the Samaritan Fund and convalescent home. There was thus a deficit between the total income and total expenditure of £3,042, but since the £2,050 for endowment of beds was not available for current expenses the excess of expenditure to be provided for was £5,092.

The accounts of the Cancer Fund were now for the first time shown separately. The ordinary income was £3,761, and the extraordinary income £4,107; of this £2,007 was from legacies, and £2,100 for the endowment of two beds. The ordinary expenditure was £5,153, and £257 was spent on a boiler. Management cost less than £85. The cost of the Cancer Investigation Department, £994, was almost covered by donations and subscriptions, £1,407 remaining in hand of the £1,605 contributed in 1899. The laboratories have been in working order since last March. Dealing with this matter at some length the report said that the work was surrounded with great difficulties, and immediate results were not to be expected. Much useful preliminary work had, however, been done already, and the investigation was being thoroughly carried out. The Board had authorised the publication of special "Cancer Laboratory Reports," in which the progress made will be recorded, and in which the results obtained in the laboratories will be detailed, and thus rendered available for the use of medical men in general. The first of these reports will be issued during the current year. Since the Board some two years ago took the initiative in the foundation of Cancer Research Laboratories, similar steps had been taken in other countries, and systematic investigation into the causation of cancer was now being carried out in Germany, under the direct auspices of the State, and in America by such bodies as The Cancer Commission of Harvard University, which had started on its work amply endowed by private munificence. It could not be doubted that tangible benefit to humanity would accrue from such concurrent work in the same direction. Since the Middlesex Hospital was able to claim the honour of having occupied the foremost position in this movement, the Board expressed confidence that the help which was being afforded abroad, either by the State itself or by private benevolence, would not be denied to the Cancer Research Laboratories, and that funds would be forthcoming so that the undertaking might not be crippled by any want of the means for carrying it out in the most thorough manner possible.

Turning next to the Convalescent Home and Samaritan Fund, the report stated that 715 patients had been received during the year, of whom 259 were men, 327 women, including 18 of the nursing staff, and 129 children. This number was rather less than in the previous year owing to the retention in the Home of male patients undergoing the open-air treatment for tuberculosis, the cure involving a stay of about six months. The board was anxious that this most useful adjunct to the hospital should afford relief to as large a number as possible; but they were so satisfied with the success of the open-air treatment, that they had resolved to continue to set aside a limited number of beds for the purpose. The ordinary income was £1,084. The ordinary expenditure was £3,848, and the extraordinary expenditure (for storage battery), £260; £3,000 was contributed from the general funds to make up the deficiency appearing under the head of extraordinary income.

The accounts of the Lady Probationer Fee Fund and Trained Nurses' Institute showed results similar to those of the previous year, a balance of £1,024 being transferred to capital. The nurses' earnings diminished slightly, amounting to £2,488, but the lady probationers' fees showed a further increase, totalling £842. The expenditure on nurses' commission had risen to £600, from the coming into operation of the increased scale, but there had been a considerable saving in the other items on the credit side of the account.

No site had yet been selected for the erection of the new laundry, nor had a scheme for centralising the boiler and engineering departments been settled upon. It had been decided to acquire No. 5 Nassau Street, adjoining the new cancer wing, and a lease for 93½ years, commencing Christmas, 1904, at £50 per annum, had been accepted.

Sir Arthur Watson moved the adoption of the report and accounts, and said they contained no sensational items, but the hospital was flourishing and everything was going on in a most satisfactory manner.

Mr. Andrew Clark, in seconding, spoke hopefully of the results to be anticipated from the Cancer Investigation Department with regard to the causation of the disease. And not only did they anticipate discoveries which might prove of the highest importance, but as they went along they were able to improve their methods of treatment in the wards. Although, in spite of a large number of cures, there were many cases that came back to them—still these recurring cases were not so many as they used to be. And in view of the statements constantly being made about vivisection, he would like to say that vivisection, in the broad acceptation of the term, was not carried on. The utmost they did was to introduce the poison or the cure into small animals such as guinea-pigs so as to observe the results.

The report was adopted, and the various officers having been re-elected, a few words from the Chairman acknowledging a vote of thanks concluded the meeting.

### ROYAL FREE HOSPITAL.

Lord Northampton presided at the seventy-third annual Court of Governors of the Royal Free Hospital on February 27th. In moving the adoption of the report he referred to the great loss sustained by that hospital and by the nation in the death of the Queen. He did not think it would be possible to put into language the sorrow felt by the nation at the loss of the best Sovereign a nation ever possessed, and by the people at large at the loss of the best friend they had ever possessed. He felt assured, however, that the King would follow in her footsteps. Turning to the affairs of the hospital he said they had made steady and consecutive progress in spite of financial difficulties. They had now a really good workable place which had been gradually brought within modern requirements, which meant that they were more alive to the necessities of hospitals. He had visited the nurses' quarters, and he saw there in some portions the cubicle system in use. To that he had a great objection, and was glad to know that efforts were being made to do away with it. The nurses should all have separate rooms and a door key, so as to have comfort and privacy. But these improvements cost money. Already they had spent some £40,000 on structural alterations and additions, and further sums were still required. Among other things lifts were urgently necessary, and he suggested the institution of a "Lift Fund," for they certainly ought to be provided. With regard to the hospital's finances, these were by no means satisfactory. The reliable income amounted only to about one third of the expenditure, and to carry on business under such conditions must be harrassing to those engaged in the struggle. It would probably be found that there was no charitable institution but was suffering from lack of funds, and this they were told was because of the military funds. But after all if charity meant that they were to divert their gifts from one object to another, then he entirely mistook the meaning of the word. There could be no charity without self-sacrifice. They had imagined that the charitable public had given extra and that old established institutions would not suffer because of the help given to the sufferers by the war. Hospitals were especially carried on for the benefit of the classes from which our soldiers and sailors were largely drawn. The public conscience wanted rousing to make amends for the want of charity shown during the past six months. The great dividing line between wealth and poverty was the question of health. To be wealthy meant, as he understood it, to have sufficient for one's wants and a little balance over, and many people considered to be wealthy would have difficulty in finding the balance over. But when sickness came the well-to-do were able to get what was best for their nearest and dearest, while the poor had the crushing feeling that they could not get what might save or prolong the lives of their sick ones. That was where the hospital came in—to bridge over that gulf between the rich and the poor, and it was for those who could to do all they could to strengthen that bridge.

Mr. Charles Burt, Chairman of the Weekly Board, seconded. They began the year, he said, with a deficit of £1,300. They worked as economically as possible, consistently with efficiency, and had got all they could by public appeals for help. During their seventy-three years they had never been in debt—had had no permanent debt—and they intended their friends should have no peace in their endeavours still to keep out. They felt it their imperative duty to keep in the very front rank, and that the expenditure they had incurred on their improvements had been well incurred. He was not sure they always gave sufficient thanks to those who did the hard, constant day and night work of the hospital—the nurses. But they had provided now separate rooms for some of them and had decided to make twenty-four more, instead of the cubicles to which the chairman had referred. Going on to another matter he said they would see from the report that it had been decided, now they had accommodation provided, that two women should be appointed as resident medical officers from May 1st. Their students were from the London Medical School for Women and for years past the appointment of properly qualified women as residents in the hospital had been under consideration. In view of this some friends of the School had promised to contribute £1,000 to the fund. It was an important step, as these were the first appointments of the kind either there or in any general hospital in London. He thought he might specially appeal to ladies everywhere to help them all they could, although they had no fashionable friends to hold bazaars and rake in £10,000 at a time.

From the report and accounts which were then adopted it appeared that the ordinary income was £6,793 and the legacies £3,526. The ordinary expenditure was £11,588, or £1,268 less than the total income. The cost of the addition to the nurses' quarters—a fourth storey having been added on each side of the central block, providing not only separate bedrooms for twenty-seven sisters and senior nurses, but also an additional sitting-room, two bath-rooms, and other conveniences—with the furnishing of the new rooms, was about £5,300, towards which nearly £3,800 had been received and promised, leaving £1,500 still to be raised. The average weekly cost of each bed was £1 8s. 10d., as compared with £1 7s. 11d. The average daily number of beds occupied was 120, as against 127. One of the two wards closed in consequence of the building operations, was reopened at the beginning of 1901, and it is hoped to reopen the other shortly, so that the 170 beds will once more be available. The number of patients treated was 36,560, as compared with 36,995 in 1899. Of these 2,030 were in-patients, 16,270 out-patients, and 18,260 casualties. The Committee reported with regret the death during the year of their colleagues Sir Charles Hall and Mr. Charles F. Greenhill, and also of Dr. John Cockle and Mr. J. Grosvenor Mackinlay, F.R.C.S. Dr. Cockle held the post of physician from 1858 until his retirement from active work in 1888, when he was appointed a consulting physician to the hospital. Mr. Mackinlay was ophthalmic surgeon from his appointment in 1880 until he resigned, on account of failing health, in March last. Mr. Henry Work Dodd, F.R.C.S., the junior ophthalmic surgeon, has been appointed ophthalmic surgeon to the hospital, and Mr. W. H. Battle, F.R.C.S., having resigned the post of surgeon, upon his appointment as surgeon to St. Thomas's, Mr. T. Percy Legg, F.R.C.S., has been elected to fill the vacancy thus caused. The report concluded with an expression of complete satisfaction by the Board with the work of Miss Brimmell, the almoner, and her assistants in the inquiry department.

On the motion of Mr. Burt, seconded by Captain Jessel, a loyal petition to the King, praying a continuance of the Royal patronage, was adopted.

The Committee of Management, with the exception of Mr. Ford North, who had resigned, and of Dr. Harrington Sainsbury, who, as a representative of the medical staff was replaced by Dr. Walter Carr, was re-elected, after which votes of thanks concluded the proceedings.

### ROYAL HOSPITAL FOR CHILDREN AND WOMEN.

The Eighty-fifth Annual Court of Governors and Friends of the Royal Hospital for Children and Women, Waterloo Bridge Road, was held at the Mansion House on February 27th, the Lord Mayor (Alderman Green) presiding.

An abstract from the report of the committee for the past year was read by the Secretary, in which it was stated that although the subscriptions and donations showed an increase over those for 1899 they were still far from sufficient to meet the annual expenditure. The accounts showed ordinary receipts of £3,474 and extraordinary receipts (legacies) £519; with ordinary expenditure of £4,355, and £494 transferred to Building Fund, the excess of expenditure over income for the year being thus £856. The opinion was expressed that the unattractive exterior of the building itself affected its claim to notice by the passer-by, but this and other disadvantages the committee confidently hoped to see remedied when the new hospital was erected; and this work they expected to be able to commence this summer. The Prince of Wales's Fund had repeatedly urged the necessity of the enlargement and rebuilding, and this year had made a grant of £250 towards the fund for the purpose. There had been several changes in the *personnel* of the committee during the year. The Rev. Geo. E. Asker, late Vicar of St. Andrew's, had resigned on his appointment to a vicarage at Tufnell Park, and the vacancy thus caused had been filled by the appointment of his successor, the Rev. G. Robinson Lees. Mr. Weatherly Phipson, in recognition of his long-continued services, had been promoted to a vice-presidency, and Mr. Frederick W. Horner, M.P. for North Lambeth, had taken his place. Mr. Geo. Herbert Phillips had likewise been made a vice-president, and was succeeded by Mr. Arthur E. Eastwood, a son of the late Treasurer, and who had recently retired from the firm who are solicitors to the institution. Mr. Fripp, who was called to the front, and had recently returned, had resigned, and Mr. H. S. Pendlebury, who had acted as *locum tenens* during Mr. Fripp's absence, had been elected to fill his place. Dr. C. Osman Bodman had been appointed resident medical officer for the ensuing year. Dr. Kenneth Steele had likewise returned from the war after being for a time invalided, and resumed his former position in conjunction with Dr. Mortimer. There had been a slight falling-off in the number of in-patients—583 as compared with 627 in 1899—though the average number of beds occupied was as usual 50. The out-patients numbered 8,379, as compared with 9,334. The average cost of each in-patient worked out at £5 18s. 10d.; of each bed, £69 5s.; and of each out-patient, 2s. 1d. Careful note appears to have been taken of the suitability for relief of the out-patients, so as to prevent abuse of the charity.

The design for the new hospital shows a building of seven floors, red-brick, faced with Doulton's terra-cotta, with open corridors for patients, and a permanent means of escape in case of fire from every ward. Although ornamental, there is to be no extravagance in construction, the object being to obtain a model children's hospital at the least possible expense. The ventilation of out-patients hall and the various wards is to be carefully secured; there will be increased accommodation for the medical and surgical staff, and operating theatres, with all the latest appliances; and as heretofore patients will be seen separately and with strict privacy.

The Chairman then moved an address of condolence to the King, with an appeal that he and Queen Alexandra will continue the patronage and support hitherto accorded. The Chairman then moved: "That the report be adopted, and that the Royal Hospital for Children and Women is in every way worthy of support, and has strong and peculiar claims upon the City of London; that the time has now arrived when it should be extended and rebuilt on modern lines; and that a subscription list be at once opened for that purpose and to free it from debt." It was a matter of gratification, he thought, that that hospital, founded so far back as 1816, still continued its good work; and a matter of congratulation that they found its present accommodation quite inadequate to its requirements. They had no alternative but to ask for aid in carrying out the extension which was enforced upon them, and he hoped the appeal would not be made in vain. It was imperatively necessary that they should have these new and enlarged buildings, but it was impossible to carry out the work without additional financial support, and he appealed to all who had the welfare of the hospitals of the metropolis at heart to combine for this particular object.

Sir Edwin Durning-Laurence in seconding the motion reminded the meeting that it was by the special permission of the present King, then Duke of Cornwall, that twenty years ago they were able to acquire the reversion of the freeholds necessary for their proposed extension. As to the need for their institution, there was more poverty south of the water than in the East End; but they were out of the way, and the appearance of their premises was against them. The hospital looked like an ordinary house in a street, and people passed by and forgot them when making their charitable dispositions. And they did not deserve this, for they had the character of doing their work in a most economic and most efficient way.

The resolution was adopted, as were also others re-electing the officers of the institution and conveying thanks to the staff for their services, and to the Lord Mayor for presiding. It was announced that subscriptions amounting to £771 had been received during the afternoon.

### EAST LONDON HOSPITAL FOR CHILDREN.

The annual Court of Governors of the East London Hospital for Children was held at the hospital on Wednesday, 27th ult. The chair was taken by Mr. H. W. Trinder.

The report for 1900 stated that the new operating theatre and four-bed ward underneath had been completed and opened; both were proving most useful. The total cost, including equipment and architects' fees, was £2,004 13s. 7d.; this had been met by two donations, amounting to £350, and by £1,384 5s. 6d. from the income of the hospital and £270 8s. 1d. out of the proceeds of the sale of the old hospital premises. This new ward was for very serious surgical cases: the annual cost was estimated at about £300. It was the first addition since the isolation block was opened in 1886.

The number of in-patients during the year was 1,541; of new out-patients 29,630, besides 6,694 casualties.

The cost per occupied bed per week was £1 4s., as against £1 4s. 6d. in 1899. This, considering the price of fuel and other necessaries, showed the care exercised by the house committee and secretary. The total receipts were £10,568 9s. 7d., only £630 of which was made up of legacies, which were lower than in any previous year. The Board trusted that future years would show an improvement. The hospital had found out of income £566 14s. for the cost of the new theatre and ward, £125 towards the maintenance of the Convalescent Home, and £45 for the Convalescent Home Building Fund. The net result of the Dickens Reading kindly given by Sir Squire Bancroft was £250 12s. 5d. The hospital's supporters were reminded that Charles Dickens was much interested in the hospital in its early days, and published an account of it under the title "A Small Star in the East" in No 30 of the *Uncommercial Traveller*. The present kitchen and rooms for the staff being now much too small, and a new mortuary and laundry, further bedrooms for the present nurses and servants, new beds for in-patients, and consequently accommodation for an increased staff being necessary, the board proposed to spend £20,000 on new buildings and extensions, as follows:—New mortuary (towards which the Prince of Wales's Fund had given £500) and new laundry with machinery, £2,500. New kitchen, casualty department (the present one being both too small and too far from the dispensary and consulting rooms of the out-patients' department); additional beds for staff, larger day accommodation for them, and accommodation for 18 additional nurses and servants required for the new wards next mentioned, £11,000; a ward of 12 beds for whooping cough, which could not at present be treated in the hospital, although one of the most fatal diseases of infancy; new wards, with 20 beds for general cases, as an additional storey on the present out-patients' department, £6,500.

The committee appealed confidently for support in carrying out the scheme—feeling justified in doing so by the excellent and increasing work (while the number of applications had increased from 6,873 in 1887 to 35,000 in 1900, the number of beds had only been increased by 17, including 10 beds in the Cromwell block for infectious cases) and by the fact that the hospital had never yet been in debt. Additional subscriptions were required for the Princess Mary Convalescent Home, which would be able to do yet more good work if it could be made self-supporting, The report closed with a paragraph from a recent issue of The Hospital on the work being done. The adoption of the report and balance-sheet having been moved from the chair, and seconded by the Rev. Father Gorman, it was carried unanimously. The rest of the business consisted of the appointment of officers.

### NATIONAL ORTHOPÆDIC HOSPITAL.

The annual meeting of the National Orthopædic Hospital, Great Portland Street, was held on Thursday, 28th ult. His Grace the Duke of Marlborough, President, was in the chair. The Duke of Marlborough said it gave him great pleasure to be able this year to preside at the annual meeting. Although he had been absent from England last year he had been in frequent communication with Mr. Tresidder, the Secretary, and Miss Hole, the Matron. He was always glad to know that satisfactory progress was being made. On the whole he thought they might congratulate themselves on progress. Last year, owing to an epidemic of scarlet fever, the hospital was closed for a month; yet 133 children and 78 adults passed through, making a total of 211 in-patients. Considering the size of the building and the limited space at disposal, he thought this undoubtedly satisfactory. Another point to which he would refer was that many soldiers had been treated: they had derived help and benefit from surgical treatment. This was the case not only with out-patients, for he had just been informed that a soldier was about to be admitted as an in-patient for treatment of a crushed foot. Many more would be coming home, and the kind of wound from which they suffered would require such treatment as was given at the orthopædic hospital. He hoped that no one would have to be sent away for want of accommodation or pressure from other sources. It was impossible not to touch on the financial condition of the hospital; this, too, was on the whole fairly satisfactory, but there was a debt of £456 in the account. It would be remembered that, acting on the advice of some members of the committee, a letter, signed by himself and Lord Farquhar, was sent to the *Times* of December 6th: this had resulted in nearly £150 so far, but there still remained about £300 to be cleared off. The ingenuity of Mr. Cooper had devised a scheme which it was greatly hoped would prove successful, viz., a matinée, to be given on March 7th. The zeal and energy of the Ladies' Committee was admirable: they had worked hard to make the December sale a success. He formally moved that the report and balance sheet be adopted.

The Rev. E. Grose Hodge seconded. He laid stress on the importance of the existing debt; if people knew of the difficulties, the sum required would not, he thought, be difficult to obtain. He believed a collection at Trinity Church had been arranged for. It was impossible in any report to tell half the good that was done. The report was unanimously adopted.

Her Grace the Duchess of Marlborough having been elected as Patron, and the Marquis of Bute and Sir Henry Harben as Vice-Presidents, the Treasurer said the existence of the debt had grieved him very much, the more so as on account of a technicality (which it was believed was now being removed) the grant from the Prince of Wales's Fund had been withdrawn. With regard to the matinée, all the boxes had been taken, and he was very sanguine of success. The position of the hospital might be summed up thus: "It does its work, but is very short of cash." Mr. I. R. Cooper proposed a vote of thanks to the Ladies' Committee, to which the Rev. E. Grose Hodge replied, and the usual votes brought the meeting to a close.

### THE WEST END HOSPITAL.

The annual meeting of the West End Hospital for Paralysis, &c. was held on Friday, 22nd ult. The adoption of the annual reports and audited accounts was moved by Mr. G. E. Porter. The medical report showed that the general health of the wards had been exceedingly well maintained; the hygienic condition had been greatly improved since the introduction of open fire-places. There had been some temporary cessation of work owing to an outbreak of measles, the cause of which was clearly traced and proved to have been unavoidable. The pressing need of the hospital was an isolation ward—the only place for suspected cases being at present in the centre of the main building, surrounded by nurses' and servants' rooms. Speaking on this point, Mr. Porter said that the plans were already drawn up by the hon. architect, Mr. G. Blizard, and the committee proposed to build an isolation ward, with ten beds, at the top of the present wards. This would release the bedroom now used for isolation purposes, and enable the committee to give up the rooms which they had been obliged to rent outside the hospital for the accommodation of some of the servants. The committee had £250 in hand (from the Prince of Wales's Hospital Fund) for the special purpose of providing a new operating room; they expected to be able to carry out the work, which would supply a long felt want, during the present year. Another scheme was a convalescent home by the sea, where the special treatment given in the hospital could be followed up; it often extended over such a lengthy period that the children had to go home for a time, in order to make room for others: this in some cases meant that the good they had received in the hospital was undone during that period. The treatment required special electrical appliances, and special skill in the massage treatment. The committee also contemplated the establishment of a nursing home, which would enable them to assist in supplying the great demand for nurses skilled in the special work for which they were trained at the hospital, as well as to provide the much needed increase of accommodation for the present nursing staff. During 1900, the number of in-patients had been 360; the number of new out-patients was 3,378; the total number of massage treatments had been 6,361, and electrical, 9,971—grand total, 16,332. The total number of out-patients' attendances had been 39,218. With regard to finance, the expenditure amounted to, for maintenance £2,839 8s. 4d., and for administration £418 0s. 8d. New subscribers were urgently needed; although, owing to the strenuous exertions of the treasurer, Captain H. A. Dowell, a large number of new supporters had come forward to help in the good work of the institution. Annual subscriptions, donations and legacies had amounted to £2,728 9s. 10d. during 1900, while liabilities amounted to £1,251 4s. at the close of the year. The committee would like to be able to clear off some of their liabilities during the current year.

In moving the adoption of the reports and accounts Mr. Porter alluded to the death of Queen Victoria. A resolution of sympathy and condolence had been placed in the minutes.

### SEAMEN'S HOSPITAL SOCIETY.

Mr. Percival Nairne presided at the eightieth annual Court of Governors of the Seamen's Hospital Society ("Dreadnought") on February 28th. He referred to the loss sustained by the hospital in the death of the Queen and of the Duke of Saxe-Coburg their President. A petition would be presented to the Duke of Cornwall asking him to accept that position in succession to his late uncle. A letter had been received from Sir Henry Burdett, as a member of the Council of the Prince of Wales's Fund, stating that that Council considered the Greenwich Hospital was no longer suitable for the work, and suggesting that the Prince of Wales's Fund and the Committee of the Seamen's Hospital should together approach the Admiralty with a view to getting it rebuilt. Mr. Huth moved the adoption of the report, which, in view of the commencement of the new century, briefly reviewed the progress of the Society since its commencement. From 1821 until 1870 the work was carried on afloat on board vessels moored off Greenwich, the average number under treatment being 160. In 1870 the old Infirmary of Greenwich Hospital was placed at the disposal of the Society, and the number of patients increased to more than 200. In 1880 a dispensary was established, in 1887 another was added, and soon afterwards the branch hospital came into being. This grew from 12 beds to the present 50—there being 225 beds at the "Dreadnought" Hospital at Greenwich—and there had now been added the London School of Tropical Medicine. During the past year the new wing of the branch hospital had been completed and was now occupied. The numbers of sick and injured seamen healed at the various establishments totalled 25,592, of whom 2,400 were in-patients. The ordinary income was £12,421, and the legacies £6,172. The ordinary expenditure was £18,915, and the extraordinary, for building improvements, &c., £354. It was with regret that the Committee stated that the piling in the foundations of the older portion of the branch hospital had been discovered to be defective, so that it would be necessary to underpin the building and put in steel piles and girders at an estimated cost of £3,000. For the outdoor treatment of consumption shelters had been erected on the rood of the Greenwich Hospital, good and promising results been reported since their opening last October. The progress of the Tropical School had exceeded the expectations of its founders. Since its opening, in October 1899, 100 post-graduate students had passed through a course of study: five of these were women graduates. Sorrow was expressed at the death of the Rev. Brooke Lambert, a member of the General Committee, and for some years Honorary Chaplain; and of Mr. Davies-Colley, for many years a surgeon on the staff, and subsequently consulting surgeon.

The report was adopted, the officers re-elected, and the customary votes of thanks brought the proceedings to a close.

### KING'S COLLEGE HOSPITAL.

The Rev. Dr. Wace presided at the sixty-second annual Court of Governors of King's College Hospital on February 28th. The report of the Committee of Management opened with a reference to the loss sustained by the hospital by the decease of her late Majesty who was its patron from its foundation in 1839 and a liberal subscriber to its funds, and recorded that at the first meeting of the committee after her death a resolution of condolence with the Royal Family was adopted and forwarded. Proceeding, the report stated that the year 1900 was one of progress and increasing usefulness, the number of in-patients admitted, viz. 2,746, being higher than in any previous year. The out-patients numbered 19,435, of which 8,242 were accidents. Not counting these latter the attendances totalled 33,718. Four hundred and seventy-eight poor women were attended at their own homes during confinement. The ordinary income was £12,466 and legacies £4,936. The ordinary expenditure was £18,933, and the extraordinary—for the convalescent home—£488. There was thus a deficit of £2,020 for the year. Annual subscriptions decreased about £120; the number of patients and the cost of fuel and food was greater; and extra expenditure was incurred in fitting up an electrical room with an X ray apparatus. Thanks were tendered to the Prince of Wales's Fund for a grant of £1,250, of which the £250 was a special donation in the hope that the tea, sugar, and butter formerly provided by most of the in-patients should in future be supplied by the hospital. It was calculated that this would increase the expenditure by from £400 to £500 a year, but the committee thought it a right step to take and all things would now be provided for the patients free. During the year three of the vice-presidents had died: the Bishop of London, Sir Richard Wilbraham, and Sir William Priestly; and the death of Professor Alfred W. Hughes had also to be recorded. Mr. Hugh Playfair, M.D., had been appointed obstetric registrar and tutor and Mr. Harrison Low, M.B., radiographer. Professor Watson Cheyne, Mr. Geo. Cheatle, and Mr. L. V. Cargill had been welcomed home from South Africa. The report was adopted; Mr. Audrey was re-elected treasurer and votes of thanks concluded the proceedings.

### THE METROPOLITAN CONVALESCENT INSTITUTION.

The Sixtieth Annual Meeting of the Governors of the Metropolitan Convalescent Institution was held yesterday afternoon at the offices, 32 Sackville Street, W., Mr. Cecil L. Wade in the chair. Among those present were Mr. M. O. Fitzgerald, Mr. C. Holmes, Mr. C. L. Lavers-Smith, Rev. J. Neal, Mr. James S. Strange, and Mr. H. P. Stebbing.

The annual report, which was read by the Secretary, Mr. Alex. Hayes, stated that last year 2,641 patients were admitted to the Walton Home, 1,558 to the Children's Branch at Broadstairs, and 1,993 to the Seaside Branch at

Bexhill, making a total of 6,192, the largest number ever received.

The accounts of the General Fund of the Institution showed an excess of expenditure over income of £1,103 and it was reported that a sum of £10,865 had been withdrawn from the small reserve fund to make up the amount required to cover the cost of the Children's Branch at Broadstairs. Reference was made in the report to the death during the year of many old friends and supporters, among them being Mrs. F. Paget, Lord Blantyre, Mr. E. H. Moscrop, and Mr. H. B. Wade, the honorary solicitor to the Institution.

Cordial thanks were accorded to the honorary medical officers, all of whom were re-appointed, and also to Messrs. Price, Waterhouse and Co., the honorary auditors; and it was announced that Viscount Portman had accepted the office of President.

Lord Haliburton and Mr. J. S. Strange were re-elected Treasurers; Mr. C. L. Wade and Mr. M. O. Fitzgerald, Chairman and Vice-Chairman respectively of the Board of Management.

### THE CANCER HOSPITAL.

Dr. Alexander Marsden, the son of the founder, presided, in the absence of Sir George Measom, the Chairman of Committees, at the fiftieth annual meeting of the Cancer Hospital, Fulham Road, on February 27th. After a resolution of condolence with the King on the death of Queen Victoria and of congratulation on His Majesty's accession to the throne, the reports and accounts were presented by Mr. F. W. Howell, the Secretary. From these it appeared that the ordinary income was £9,011, and the legacies £7,484. The ordinary expenditure was £11,859 and the extraordinary expenditure £1,853, of which £1,567 was for erecting and furnishing extra sleeping accommodation for the nursing staff. This building was in a quiet part of the grounds, and had been found of the greatest comfort. The receipts, as compared with 1899, showed a decrease of £5,829, the legacies being less by £6,256, but subscriptions and donations showing a rise of £524. During the year the average daily occupation of beds was 88. There were 725 new in-patients and 1,640 new out-patients, with 13,638 attendances. Mr. W. M. S. Robinson had been appointed senior house surgeon in place of Mr. R. G. Murray, who had gone to the war. Mr. E. Z. Davies had succeeded as junior house surgeon. The death is reported of Admiral Ward, a member of the Board since 1895. On the motion of the Chairman, seconded by General Bailie, the report was adopted, and the meeting closed with the customary votes of thanks.

### CENTRAL LONDON OPHTHALMIC.

The fifty-seventh annual meeting of the Central London Ophthalmic Hospital was held on February 28th, Mr. Stanford Burrons presiding. The report presented stated that the number of patients admitted was 12,538. Of these 338 were taken into the wards. The number of operations was 944, and the attendances totalled 31,440. The income for the year was £1,698, and the expenditure £1,788, including £40 for repairs, the excess of expenditure over income being thus £90. For the building fund £353 was received during the year, making the balance to credit of that fund £3,000. The committee remind governors and friends that the plans for the rebuilding so urgently required cannot be commenced until a minimum of £5,000 has been obtained, as without that it would be useless to apply for a renewal of the lease. Dr. C. O. Hawthorne has been elected to the post of physician to the hospital. The report was adopted. Mr. T. Brittin Archer, responding to a vote of thanks to the medical staff, said he felt assured the hospital would only be rebuilt by the efforts of the governors in enlisting the support of their friends.

### BROMPTON HOSPITAL.

The Duke of Wellington presided at a quarterly court of the governors of the Hospital for Consumption, Brompton, on February 28th. The report read by the secretary stated that plans were being prepared for the proposed Country Branch and Convalescent Home to be erected at Heatherside, near Bagshot, funds for which are needed. A vote of condolence with the King and Queen on the death of Her late Majesty and of congratulation on their accession had been passed by the Board. Mr. Lewis Karslake had resigned the post of surveyor, which he had held for nearly twenty years, and had been succeeded by Mr. Alexander Forrester, who had been associated with him in his office. The number of patients reported as having been admitted since the last court was 327. Of these 284 had been discharged, many greatly benefited, while 52 had died. The new out-patient cases numbered 3,142. Several legacies were mentioned, among them being £10,000 from Mr. Samuel Lewis, payable after the death of the widow. The proceedings were very brief, and closed with a vote of thanks to the chairman.

